# Frontiers and hotspot evolution in research on Alzheimer’s disease and hypertension: a bibliometric analysis from 2004 to 2023

**DOI:** 10.3389/fneur.2025.1514054

**Published:** 2025-04-28

**Authors:** Wenying Liu, Yaya Zhao, Yutong Rao, Zijing Wu, Yun Peng, Lianggeng Gong

**Affiliations:** ^1^Department of Radiology, The Second Affiliated Hospital, Jiangxi Medical College, Nanchang University, Nanchang, China; ^2^Jiangxi Provincial Key Laboratory of Intelligent Medical Imaging, Nanchang, China

**Keywords:** Alzheimer’s disease, hypertension, bibliometrics, Web of Science, CiteSpace, VOSviewer

## Abstract

**Background:**

Alzheimer’s disease (AD) is a neurodegenerative disease that imposes a heavy burden on patients and their families. Hypertension is an important risk factor for AD, but the specific mechanism of its impact is still unclear. This study thus aimed to analyze the relationship and trend changes between AD and hypertension through bibliometric methods.

**Methods:**

Literature on AD and hypertension was retrieved from the Web of Science Core Collection (WoSCC) database between 2004 and 2023. Data regarding countries, institutions, authors and journals were sourced from WoSCC. CiteSpace and VOSviewer were used for data visualization, including author collaboration, timelines view of references, reference bursts and overlay visualization maps of keywords. Excel 2018 software was used for the statistical analysis.

**Results:**

A total of 1,833 publications were ultimately included. From 2004 to 2023, the number of publications per year basically showed an increasing trend. The United States (United States) not only had the largest output of publications and the highest H-index but also had the seven highest frequencies of publication institutions. Kehoe, Patrick ranked first with the most articles among 9,330 authors. The journal with the most published articles was the Journal of Alzheimer’s Disease. Reference analysis revealed a hotspot in the exploration of the pathophysiological association between AD and hypertension. Second, the treatment effects and potential risks of antihypertensive drugs (AHDs) on AD are also the focus of research. Researchers have carried out a series of studies ranging from basic research to clinical research on AHDs for the treatment of AD. Finally, personalized treatment strategies will also become one of the hotspots of future research. Controlling hypertension through lifestyle changes and medication interventions in AD patients is a promising strategy. The analysis of keywords revealed that “amyloid deposition,” “preeclampsia,” “Corona Virus Disease 2019 (COVID-19)” and “biomarkers” have been research hotspots in recent years.

**Conclusion:**

By analyzing the references and keywords, we summarized the hot topics and research trends in this field. These findings provide useful information for researchers to explore the relationship between hypertension and AD further, with the hope of providing more effective treatments for AD patients to delay disease progression and improve quality of life.

## 1 Introduction

Alzheimer’s disease (AD) is an insidious progressive form of neurodegenerative dementia that is typically characterized by memory impairment, cognitive impairment, and limitations in daily activities (1). It is one of the leading age-related brain diseases in older adults, and the fifth leading cause of death in the adult population, which has a strong impact on the physical and mental health and quality of life of patients and their families (2). Its specific pathogenic mechanism is not completely clear. The primary pathological hallmarks of AD include the deposition of β-amyloid (Aβ) within the brain, the development of neurofibrillary tangles resulting from the abnormal phosphorylation and aggregation of tau protein in nerve cells, and the subsequent damage to and death of neurons (3). Cholinesterase inhibitors and antagonists of N-methyl-D-aspartate receptors (4, 5) have been approved for managing the symptoms of AD. According to reports, hypertension, particularly in midlife, is an important risk factor for the development of AD (6, 7). Hypertension accelerates microvascular injury in Aβ-induced AD, ranging from cerebral microhemorrhages to blood–brain barrier (BBB) disruption and subsequent neuroinflammation (8, 9). Clinical trials have indicated that the use of antihypertensive drugs (AHDs) to lower blood pressure can generally enhance cognitive function (10). In addition, researchers have reported that AHDs have significant effects on neuroprotective therapy (11). However, the specific mechanism by which hypertension affects AD is still unclear.

Understanding the relationship between hypertension and AD can increase our understanding of the pathophysiological mechanisms underlying AD, which is highly important for improving and delaying AD progression by controlling blood pressure. Although many studies on hypertension and AD have been conducted, the research trends and dynamic changes in this field are still elusive. Bibliometrics offers statistical analysis and evaluation of literature, including a range of indicators such as document count, citation frequency, author collaboration networks, and journal impact. It is capable of analyzing development and research outcomes within specific disciplines or fields (12). Scholars have employed bibliometric methods to clarify the relationship between AD and other conditions, including epilepsy, sleep and vitamins (13–15), but none of these studies have explored the relationship between AD and hypertension.

Therefore, we aimed to reveal the research trends and dynamic changes in this field for relevant scholars via bibliometric analysis of hypertension and AD, which might promote research on the mechanism and treatment of AD.

## 2 Methods

### 2.1 Search strategy

**Figure 1:**
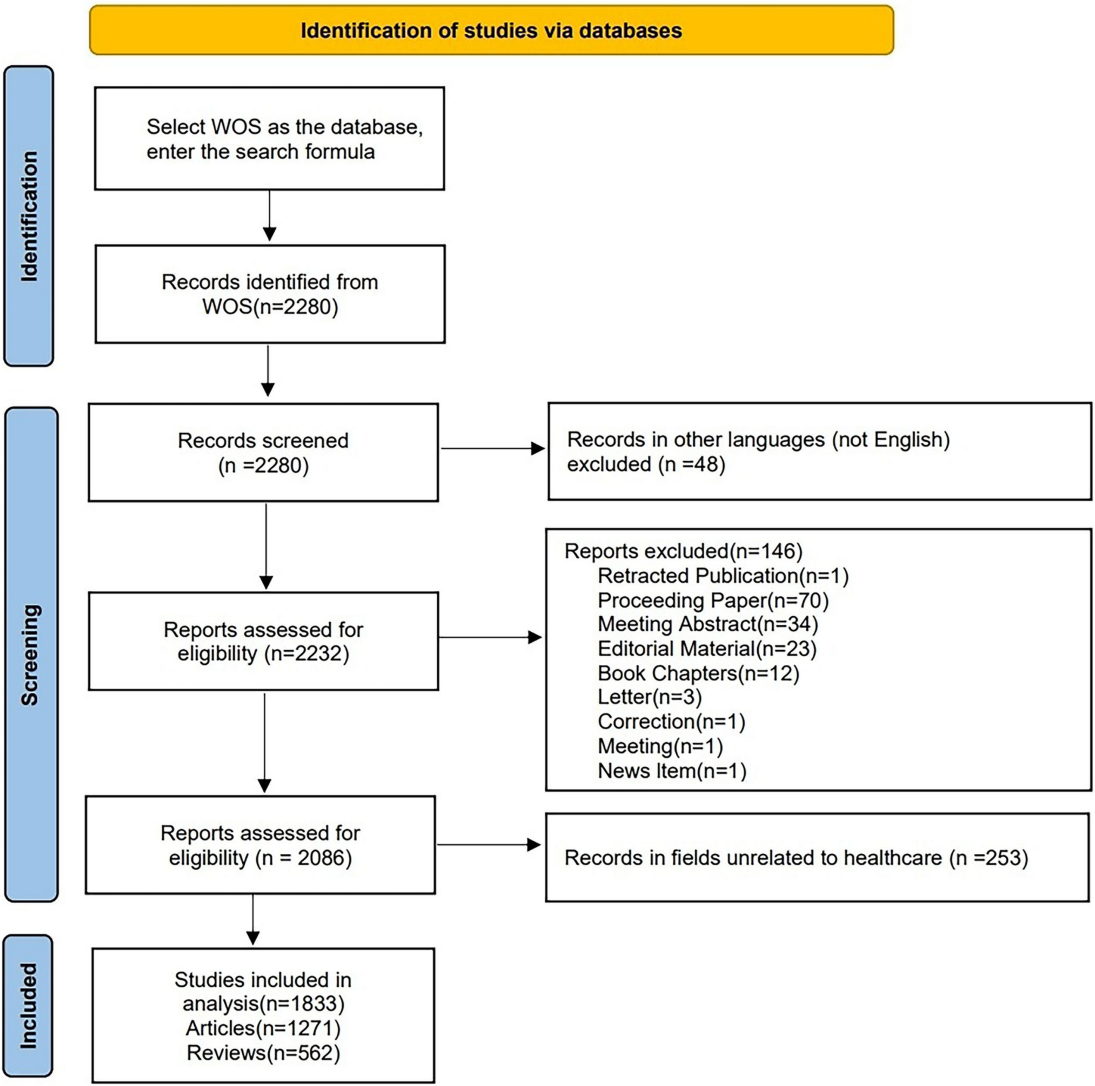
Flowchart of the publication screening process.

On July 5, 2024, we retrieved all the literature related to hypertension and AD from the Web of Science Core Collection (WoSCC). The search strategy was as follows: TS = ((“Alzheimer’s” OR “Alzheimer’s disease”) AND (“hypertension” OR “high blood pressure” OR “hypertensive” OR “hypertension”)) AND FPY = (2004–2023). To reduce bias further, we limited the search to articles and reviews in English, with Figure 1 illustrating the specific flow chart. Ultimately, 1,833 studies were retrieved and exported in plain text format, which included complete records and references.

### 2.2 Data analysis and visualization

Excel 2018 was used to display the trends in the number of articles published by year. Visualization analysis of the references was completed via CiteSpace (v.6.3.R4). VOSviewer (v.1.6.20) was used to analyze the number of articles published by each author and the keyword co-occurrence. Additionally, in an overlay visualization map of keywords, the size of the nodes represents the number of keyword occurrences. The H-index is used to evaluate the amount and level of academic output of researchers and journals (16).

## 3 Results

### 3.1 Publication trends

**Figure 2:**
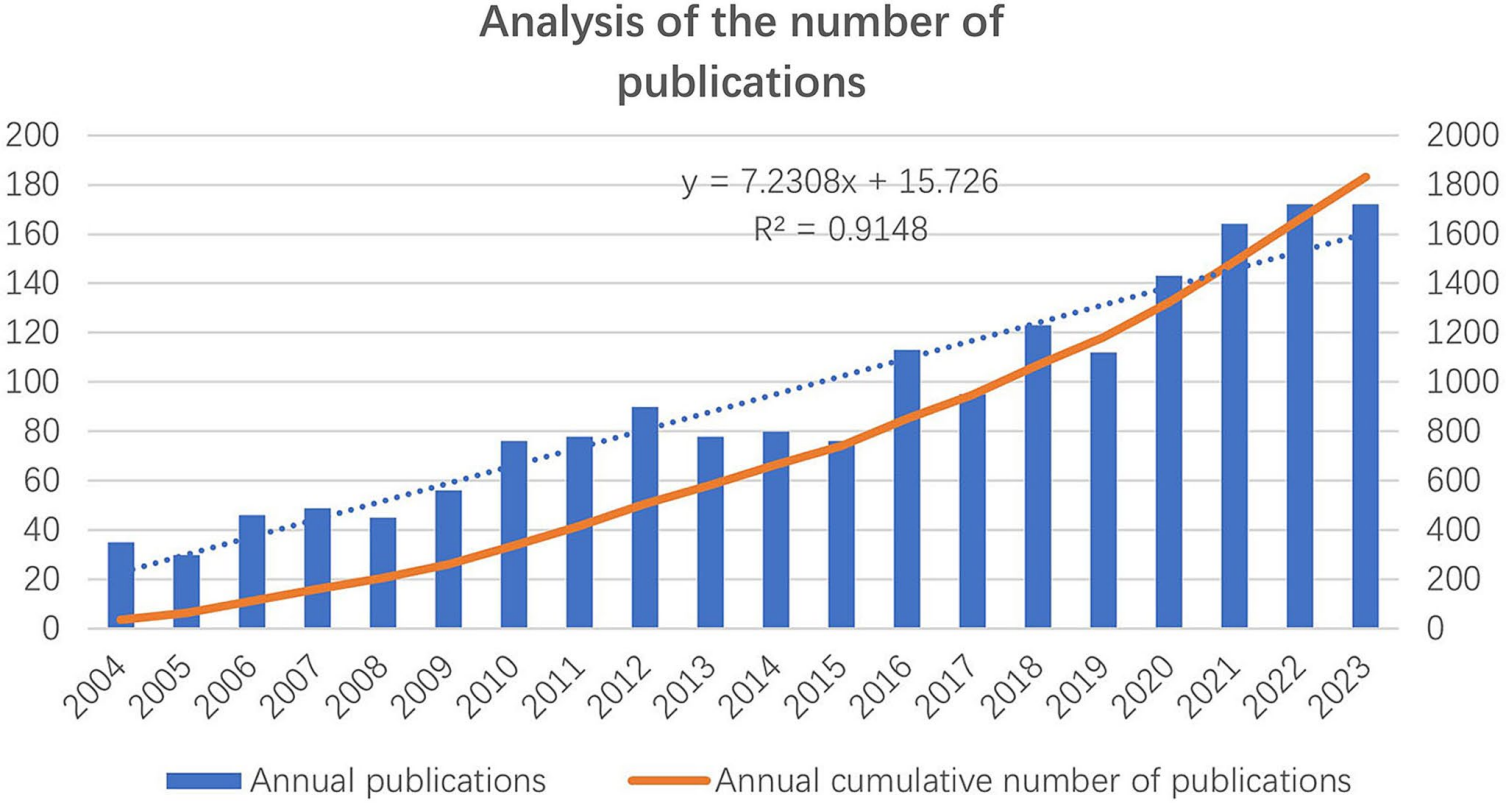
The number of annual publications and the annual cumulative number of publications from 2004 to 2023.

Figure 2 shows the growth trend of the number of annual publications and the annual cumulative number of publications from 2004 to 2023. The number of annual publications has shown an increasing trend, with over 150 publications consistently published from 2021 to 2023.

### 3.2 Analysis of countries and institutions

Out of 90 countries, 56 have satisfied the criterion of a minimum document count of 3. In accordance with Table 1, among all the countries where the literature was published, the top three in terms of the number of publications were the United States of America (USA) (727), China (200) and England (176). Among these countries, the top three in terms of citations to the literature were the USA (53,553), England (23,967) and China (7,280). The top three countries in terms of the H-index were the USA (100), England (52), and Italy (43). An analysis of publication, citations to the literature and the H-index indicated that the USA, England, China and Italy were the main research powers in this field.

**Table 1:** The top 10 productive countries.

Rank	Countries	Record count	H-index	Times cited
1	USA	727	100	53,553
2	China	200	39	7,280
3	England	176	52	23,967
4	Italy	111	43	6,050
5	Japan	110	38	4,425
6	Australia	94	39	7,027
7	Canada	89	31	5,434
8	Netherlands	87	33	7,075
9	South Korea	80	23	1,661
10	France	79	31	5,399

Among the 2,646 institutions, 468 satisfied the criterion of a minimum document count of 3. Table 2 lists the top 10 most productive institutions. The top three in terms of the number of publications were University of California system (101), Harvard University (59) and the Department of Veterans Affairs (*n* = 58). The top three in terms of citations to the literature were the University of California system (11,453), the University of London (6,977) and the Karolinska Institute (6,388). The top three institutions in terms of the H-index were the University of California system (38), the Department of Veterans Affairs (31) and the Veterans Health Administration (31).

**Table 2:** The top 10 most productive institutions.

Rank	Institution	Country	Count	Times cited	Average per item	H-index
1	University of California System	USA	101	11,453	113.4	38
2	Harvard University	USA	59	4,242	71.9	26
3	Department of Veterans Affairs	USA	58	6,350	109.48	31
4	Veterans Health Administration	USA	56	6,336	113.14	31
5	University of Texas System	USA	50	1,675	33.5	24
6	Institut national de la santé et de la recherche médicale	France	47	3,741	79.6	22
7	University of London	England	45	6,977	155.04	26
8	Karolinska Institute	Sweden	44	6,388	145.18	26
9	University of Washington	USA	37	2,915	78.78	23
10	Harvard Medical School	USA	35	2,160	61.71	21

### 3.3 Analysis of authors and cited authors

**Figure 3:**
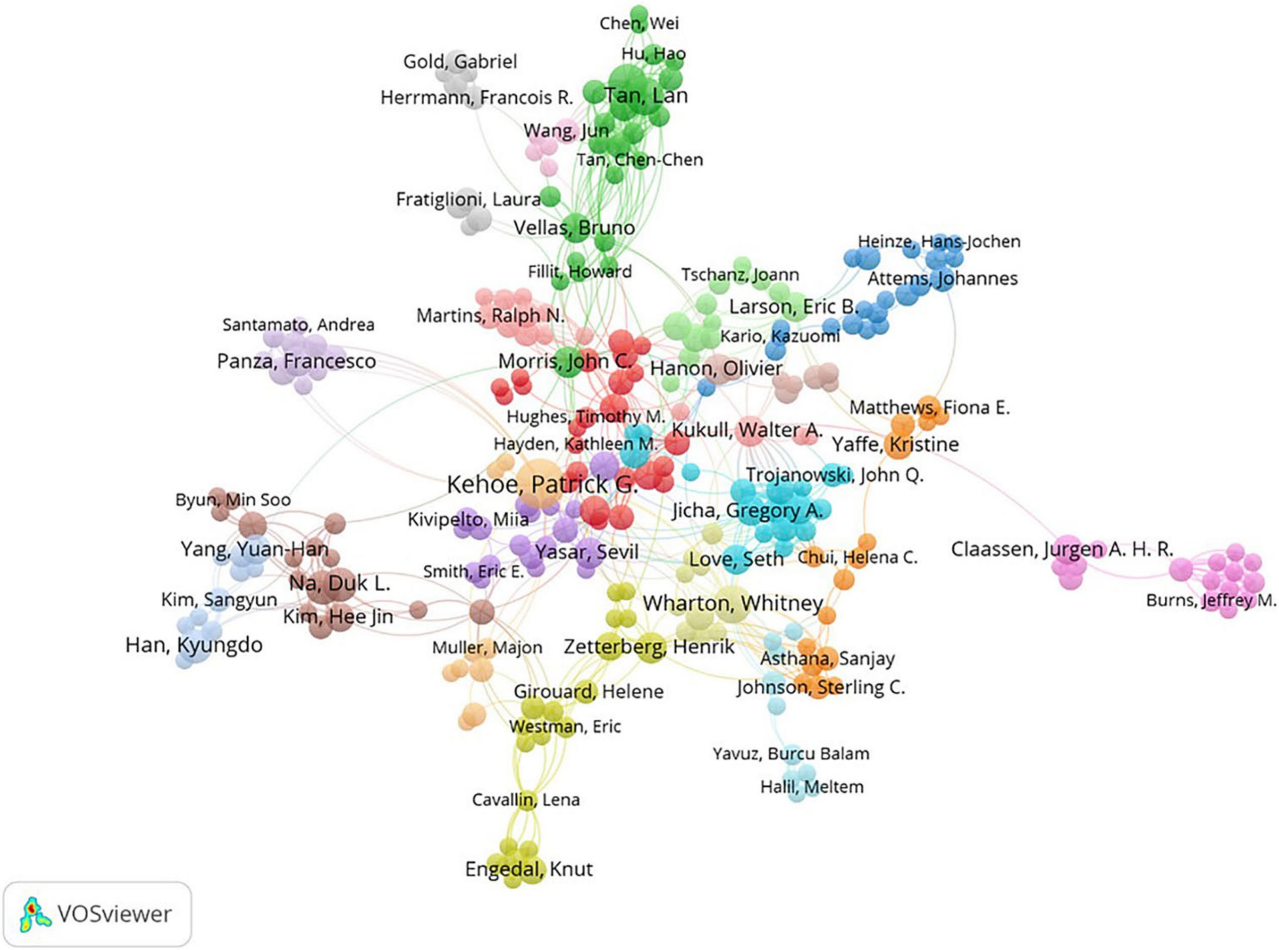
Visualization of author collaboration.

Among 9,330 authors, 416 satisfied the criterion of a minimum document count of 3. The top 10 most productive authors and the most cited authors are listed in Table 3. In terms of publication volume, Kehoe, Patrick (28 publications, H-index = 56) from England ranked first, followed by Wharton, Whitney (13 publications, H-index = 27) from the USA and Yu, Jin-Tai (13 publications, H-index = 89) from China. Kivipelto, M was the author with the highest number of citations and was cited 416 times in total. Figure 3 shows the visualization of cooperation among authors. The cooperation between these authors was not very close.

**Table 3:** The top 10 most productive authors and the most cited authors.

Rank	Author					Cited author		
	Content	Count	Country	H-index	Average per item	Content	Citations	Country
1	Kehoe, Patrick	28	England	56	69.87	Kivipelto, M	416	England
2	Wharton, Whitney	13	USA	27	54.33	Skoog, I	381	Sweden
3	Yu, Jin-Tai	13	China	89	37.7	Iadecola, C	377	USA
4	Winblad, Bengt	12	Sweden	145	66.78	Qiu, Cx	346	China
5	Hanon, Olivier	12	France	46	40.67	Launer, Lj	316	USA
6	Tan, Lan	12	China	79	37.14	Luchsinger, Ja	270	USA
7	Han, Kyungdo	12	South Korea	50	11.41	De La Torre, Jc	261	USA
8	Panza, Francesco	10	Italy	71	81.27	Forette, F	260	France
9	Scheltens, Philip	10	Netherlands	148	96.39	Mckhann, G	249	USA
10	Reddy, P. Hemachandra	10	USA	78	72.14	Kalaria, Rn	234	England

### 3.4 Analysis of published journals

The top 10 journals in terms of publications are listed in Table 4. The top three in terms of the number of publications were the Journal of Alzheimers Disease (*n* = 199), Alzheimers & Dementia (*n* = 61) and Frontiers in Aging Neuroscience (*n* = 51). The IFs (2023) of the top 10 most productive journals ranged from 1.8 to 13, which indicated a significant difference in their influence. In addition, the categories of these journals were mainly clinical neurology and gerontology.

**Table 4:** The top 10 most productive journals.

Rank	Journal	Publications	Countries	JCR	IF(2023)	Category
1	Journal of Alzheimers Disease	199	Netherlands	Q3	3.4	Neurosciences
2	Alzheimers and Dementia	61	USA	Q1	13	Clinical neurology
3	Frontiers in Aging Neuroscience	51	Switzerland	Q2	4.1	Gerontology
4	Current Alzheimer Research	39	Netherlands	Q3	1.8	Clinical neurology
5	Neurobiology of Aging	34	England	Q2	3.7	Gerontology
6	PLoS One	34	USA	Q1	2.9	Multidisciplinary science
7	Dementia and Geriatric Cognitive Disorders	29	Switzerland	Q3	2.2	Clinical neurology
8	Journal of the American Geriatrics Society	27	USA	Q1	4.3	Gerontology
9	Alzheimers Research and Therapy	24	England	Q1	7.9	Clinical neurology
10	International Journal of Geriatric Psychiatry	23	England	Q2	3.6	Gerontology

### 3.5 Analysis of references

**Figure 4:**
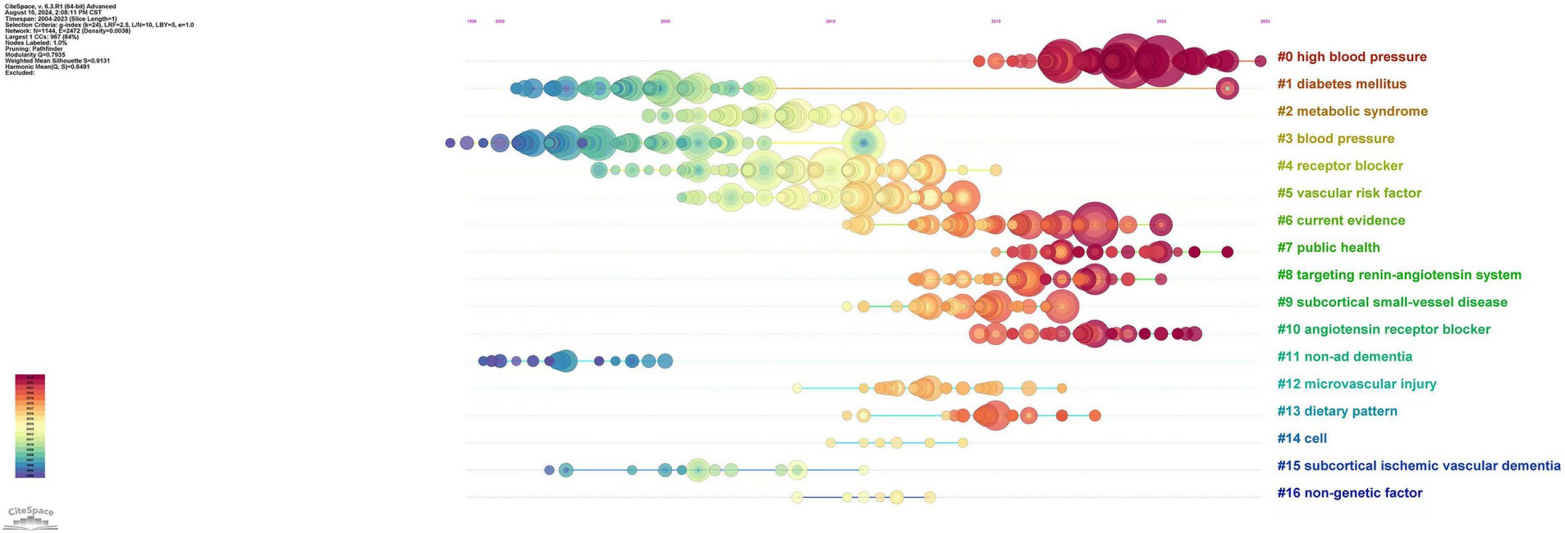
Timeline of reference citations on the relationship between hypertension and AD. Clusters are numbered from #0.

A total of 17 major clusters were generated from the reference citation network diagram after cluster analysis. The clustering module value (Q) was 0.7935 > 0.3, indicating that the cluster structure was significant, and the average clustering contour value (S) was 0.9131 > 0.7, indicating that clustering was reliable and satisfactory. As shown in Figure 4, the clusters arranged from top to bottom are #0 high blood pressure, #1 diabetes mellitus, #2 metabolic syndrome, #3 blood pressure, #4 receptor blocker, #5 vascular risk factor, #6 current evidence, #7 public health, #8 targeting renin-angiotensin system, #9 subcortical small-vessel disease, #10 angiotensin receptor blocker, #11 non-ad dementia, #12 microvascular injury, #13 dietary pattern, #14 cell, #15 subcortical ischemic vascular dementia and #16 non-genetic factor. The earliest cluster focused on diabetes mellitus, receptor blockers and subcortical ischemic vascular dementia. Clusters focused on high blood pressure and angiotensin receptor blockers have become increasingly popular in recent years. According to the timeline, it can be speculated that the current frontiers in this field are the clinical application of antihypertensive drugs in AD, with the research focus gradually shifting from the pathophysiological mechanisms of AD and hypertension to specific clinical applications.

For nearly 20 years, scholars have carried out a series of studies ranging from experimental to clinical applications concerning the relationship between hypertension and AD. As shown in Supplementary Table 1, the main research content of the top 25 papers on outbreak intensity has been collated for better understanding. The strength of the citation bursts of the top 25 papers ranged from 11.68 to 28.22.

### 3.6 Analysis of keywords

**Figure 5:**
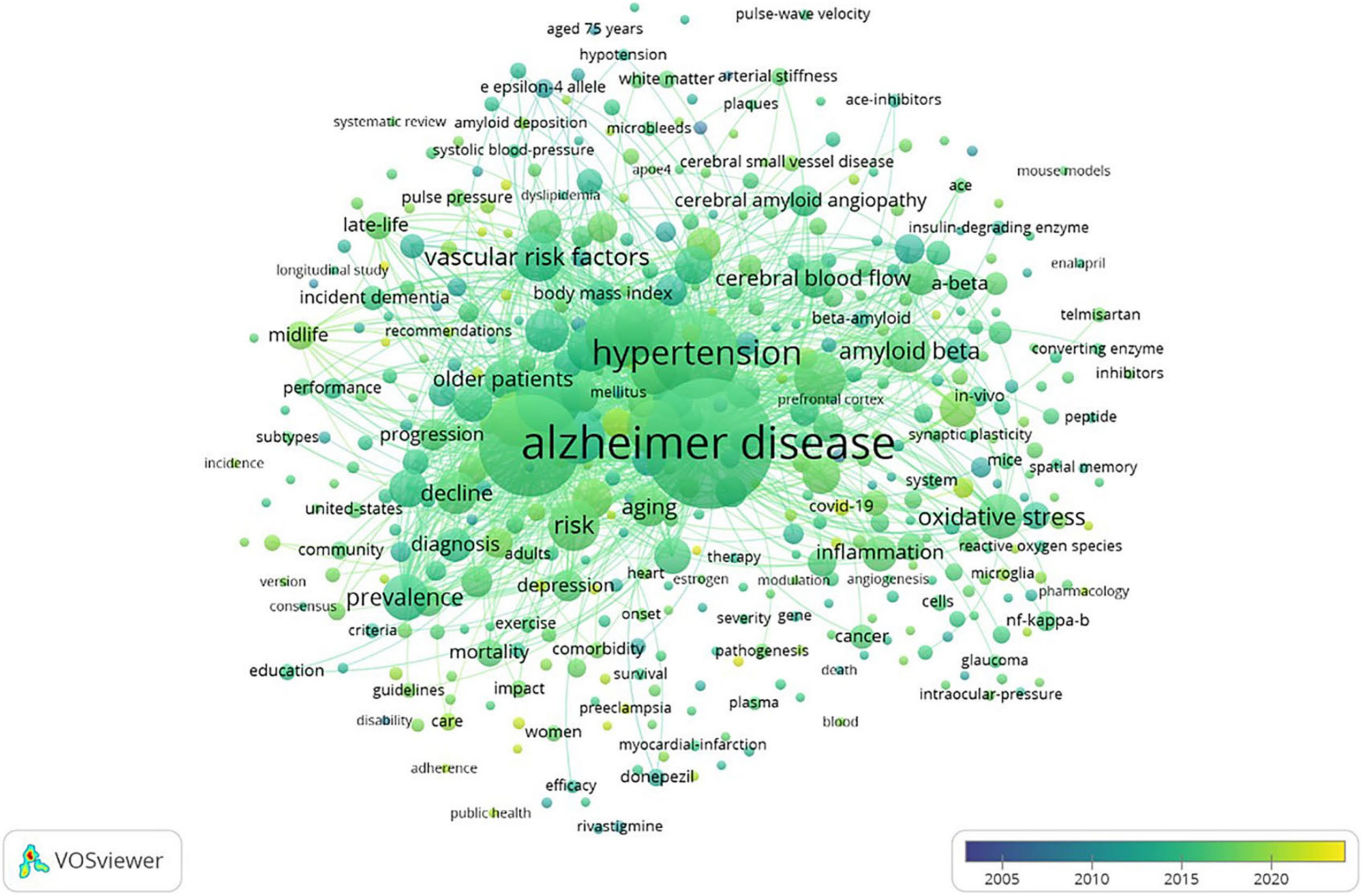
Overlay visualization map of keywords on VOSviewer. The color represents the average time of occurrence, and a color closer to yellow indicates a time closer to the present.

Among the 7,414 keywords, 445 satisfied the criterion of a minimum document count of 7. As shown in Table 5, the top five keywords for frequency and TLS were Alzheimer’s disease (1,208 times), dementia (742 times), hypertension (547 times), blood pressure (320 times), and risk factor (298 times). “Amyloid deposition,” “preeclampsia” and “COVID-19” have appeared in recent years, as shown in the overlay visualization map of keywords in Figure 5.

**Table 5:** The top 20 most frequent keywords.

Rank	Keywords	Occurrences
1	Alzheimer disease	1,208
2	Dementia	742
3	Hypertension	547
4	Blood pressure	320
5	Risk factor	298
6	Mild cognitive impairment	259
7	Cognitive dysfunction	212
8	Association	206
9	Vascular dementia	204
10	Brain	195
11	Risk	192
12	Vascular risk factors	169
13	Diabetes	157
14	Prevalence	152
15	Oxidative stress	146
16	Cognitive decline	144
17	Amyloid beta	142
18	Cognitive function	137
19	Decline	125
20	Stroke	116

## 4 Discussion

This study collected 1833 studies on hypertension and AD over the past two decades and evaluated them via bibliometrics method. According to this study, publications related to AD and hypertension research have shown an increasing trend over the past two decades. Additionally, the number of articles published in the past 3 years has remained above 150, which suggests that interest in the research field remains strong. This phenomenon may be related to the increases in hypertension and AD incidence rates with increasing age (17). Among the top 10 most productive countries that published the most publications, the USA not only had the largest output in publications but also had the first H-index ranking. Moreover, seven of the top 10 institutions in terms of productivity were from the USA, indicating that this country has made significant contributions in this field. Although China ranked second in terms of publication frequency, it lacked influential institutions. China should focus on developing influential research institutions and strengthening international cooperation in the future. Author analysis revealed a network of core author collaborations in the field of hypertension and AD research. The cooperation among authors presented regionalization, with relatively less international cooperation. Therefore, it is necessary to strengthen the international cooperation of authors and seek better academic cooperation. By analyzing the journals about hypertension and AD, we determined that researchers have concentrated on the fields of neuroscience and geriatrics. Among these journals, the Journal of Alzheimers Disease (*n* = 199) was the most productive journal, and Alzheimers & Dementia (*n* = 61) was the second most productive journal in this field, with an impact factor of 13 from Q1 journals, which indicated that the field is currently a research hotspot.

The strongest citation burst was from an article published by Livingston et al. (18) in the journal Lancet entitled “Dementia prevention, intervention, and care: 2020 report of the Lancet Commission.” This paper provides sufficient new evidence to support the addition of three additional modifiable risk factors for dementia to the 2017 Commission model (excessive alcohol, traumatic brain injury, and air pollution). They added the latest evidence on the nine risk factors implicated in the 2017 Commission (education, hypertension, hearing impairment, smoking, obesity, depression, inactivity, diabetes, and social contact). The updated 12-risk factor life-course model of dementia prevention provides comprehensive prevention, intervention, and care guidance for dementia patients, which will vastly improve living and dying for them and their families. Reports have indicated that midlife hypertension, defined as hypertension at 40 years of age, is associated with reduced brain volume and increased white matter hyperintensity volume but not amyloid deposition (19). Blood pressure decreases later in life, which is related to the development of dementia and may be caused by the development of dementia (20, 21). Four meta-analyses of blood pressure medications used to lower high blood pressure suggested reduced dementia in those in the intervention group for outcomes of any dementia as well as clinically diagnosed AD (22–25). Additionally, amyloid imaging detected amyloid in the brain with high sensitivity and specificity in people with AD when the gold-standard comparison was either neuropathology or clinical diagnosis, distinguishing AD from other neurodegenerative conditions (26).

Dementia is a progressive and typically irreversible deterioration of cognitive function that is most often observed in older adults. Vascular dementia is the second most common cause of dementia after AD and the most common cause of nonneurodegenerative dementia (27). Among vascular risk factors, chronic arterial hypertension is a major contributor to cognitive impairment (28). Hypertension was defined as a systolic blood pressure ≥ 140 mmHg or diastolic blood pressure ≥ 90 mmHg (29), which is the most important risk factor for cerebrovascular disease. The American Heart Association has discussed the specific role of vascular risk factors in cognitive impairment in previous statements (28). Unlike vascular dementia, which involves mainly cognitive impairment caused by cerebrovascular disease, brain tissue ischemia and hypoxia, and neuronal damage (30), hypertension may exacerbate the potential mechanisms of AD progression, including damage to the structure and function of the brain microcirculation, disruption of the BBB, increased neuroinflammation and oxidative stress, and impaired clearance of Aβ (31).

On the basis of the reference citation timeline and analysis of the top 25 references, we summarized the research trends in the relationship between hypertension and AD, with a focus on the following three aspects:

First, studies have focused on the pathophysiological association between AD and hypertension. Research on the vascular hypothesis and amyloid cascade hypothesis of AD pathogenesis has revealed that hypertension plays an important role in the pathogenesis of AD. As age increases, the functional and structural adaptation of cerebral arteries to hypertension can be impaired, which may lead to a significant decrease in fluid dynamic resistance in the proximal arteries, placing a heavy burden on the downstream part of the cerebral microcirculation and causing microvascular damage (32). In addition, the oxidative stress induced by stress intensifies. Higher pressure results in increased wall tension-related cellular stretch, which promotes oxidative stress in endothelial cells and vascular smooth muscle cells by inducing and upregulating nicotinamide adenine dinucleotide phosphate hydrogen oxidants (33) and increasing the mitochondrial production of reactive oxygen species (34). This may lead to BBB destruction, neuroinflammation, and white matter damage, promoting neuronal damage, amyloid plaques, and cerebral amyloid angiopathy (28). The resulting oxidative stress leads to structural damage to endothelial cells, pericyte injury and increased activation of matrix metalloproteinases (35, 36). Increased matrix metalloproteinase activity leads to disruption of tight junctions and breakdown of the extracellular matrix, resulting in damage to the BBB. The BBB is a functional part of the neurovascular unit that separates the central nervous system from the circulation. The damaged BBB allows IgG, hemoglobin, fibrinogen, and highly inflammatory pathogen-associated molecular patterns in the plasma to enter the brain parenchyma, activating microglia and promoting neuroinflammation, leading to neuronal apoptosis and impaired synaptic function (37, 38). Hypertension is associated with transverse aortic constriction and enhances Aβ deposition. Aβ deposition can be detected in the mouse brain 4 weeks after hypertension is induced (39). In transgenic mice infused with angiotensin II, angiotensin II administration increased the activity of β-secretase and the cleavage rate of the Aβ protein precursor, leading to the accumulation of Aβ in blood vessels (40). These findings validate the key role of hypertension in the pathogenesis of AD. However, the mechanism by which hypertension may lead to AD remains to be studied.

Second, studies have focused on AHDs, particularly calcium channel blockers, angiotensin-converting enzyme inhibitors, angiotensin II receptor blockers, and β-blockers (BBs), for their role in mitigating AD-related pathology and their preventive effects. Studies indicate that reducing blood pressure, influencing the renin–angiotensin system, and/or modulating intracellular calcium homeostasis may offer protection against neural damage resulting from vascular changes, increased permeability of the BBB and inflammation (41, 42). Law CSW et al. summarized the latest AD-related observations of four groups of 11 AHDs in preclinical studies and proposed that angiotensin II receptor blockers may have the best potential for treating AD (41). Angiotensin II receptor blockers can control AD by reducing neuroinflammation, increasing the clearance rate of Aβ, and/or reducing the level of hyperphosphorylated tau protein (43–45). Calcium channel blockers have mostly failed to demonstrate good efficacy in trials (46, 47), whereas angiotensin-converting enzyme inhibitors might prevent the conversion of neurotoxic Aβ_42_ to Aβ_40_, increasing the risk of IQ deterioration (48). BBs have lower potency than other AHDs do, in addition to poor penetration across the BBB (49). Although there is strong and concrete evidence that AHDs are useful in the management of AD, the efficacy of AHDs seems to be limited by the age of patients. Vazirinejad et al. suggested that only AD patients aged 40 years and above presented better cognitive performance (50). However, this may not be a handicap, as AD mostly affects people aged over 65 years. In addition, another potential area to explore is the use of AHDs in conjunction with the currently approved AD drugs, which is expected to lead to improved treatment and prognosis for AD patients.

Finally, the pathogenesis of AD is multifactorial and involves genetic susceptibility and environmental interactions, among which hypertension is the most decisive factor among all modifiable risk factors mediating the development of AD (51). Controlling hypertension through lifestyle changes and medication interventions in AD patients provides promising strategies to alleviate these effects.

Keywords analysis revealed that “amyloid deposition” “preeclampsia” “COVID-19” and “biomarkers” have emerged in recent years and can be considered new research directions in the fields of hypertension and AD. Postmortem studies have revealed that hypertension is related to several features of AD including increased Aβ plaque burden and neurofibrillary tangles (52). In animal models of preeclampsia intracranial cerebral arteries and hippocampal arterioles do not remodel and have impaired myogenic tone in response to pregnancy which may lead to hyperperfusion of the brain increase permeability of the BBB and cause neuroinflammation (53). Studies have shown that a history of preeclampsia is associated with an increase in white matter volume and a decrease in gray matter volume which is usually related to cognitive decline (54–57). Research has also shown that women with preeclampsia have a greater risk of stroke which increases their risk of developing mixed dementia caused by AD and vascular factors (58, 59). AD and COVID-19 have common risk factors including age hypertension diabetes cardiovascular disease and the presence of the apolipoprotein E *ε* 4 allele (60, 61). A study comparing 5,128 AD-positive patients with 431,695 AD-negative patients revealed that a pre-existing diagnosis of AD predicts a higher risk of COVID-19 and mortality among elderly adults. Moreover the prescription of angiotensin II receptor blockers was significantly associated with a lower risk of COVID-19 occurrence among AD patients (62). George et al. reported that early adult hypertension was associated with differences in late-life neuroimaging biomarkers such as smaller cerebral cerebral gray matter hippocampal frontal cortex and parietal cortex volumes; larger lateral ventricles third ventricles and free water volumes; and lower fractional anisotropy (63). These biomarkers are associated with cognitive decline and dementia. The above research results indicate that there are still many new directions worth exploring in the fields of AD and hypertension, which may provide new ideas for exploring the pathogenesis and treatment of AD.

## 5 Limitations

There were several limitations in this study. First, all English articles and reviews were retrieved and downloaded from the WoSCC, so they may not represent the complete research field of the relationship between hypertension and AD. Second, this study analyzed only the overall situation over the past 20 years. Finally, factors such as self-citation may have led to research bias.

## 6 Conclusion

To our knowledge, this is the first bibliometric analysis of hypertension and AD. This study revealed that countries in America and Europe, especially the USA, have advantages in terms of publication and research cooperation in terms of the relationship between hypertension and AD. Asian countries, such as China and Japan, need to actively seek international cooperation to increase their global influence on further development in this field. Analysis of references and keywords indicated that research in this field has shifted from the pathophysiological relationship between AD and hypertension to clinical research. The therapeutic effects and potential risks of AHDs on AD have also been research hotspots in recent years. Additionally, new research areas such as “amyloid deposition,” “preeclampsia,” “COVID-19,” and “biomarkers” may offer new insights into exploring the pathogenesis and treatment of AD. We believe that these findings will provide useful information for researchers to further explore the role of hypertension in AD.

## Data Availability

The original contributions presented in the study are included in the article/Supplementary material, further inquiries can be directed to the corresponding author.
