# Supplementary material for: Frontiers and hotspot evolution in research on Alzheimer’s disease and hypertension: a bibliometric analysis from 2004 to 2023
Source: Front Neurol. 2025 Apr 28;16:1514054. doi: 10.3389/fneur.2025.1514054 (PMC12066472; doi:10.3389/fneur.2025.1514054)
Supplement: Supplementary file 1 [file Table_1.docx]

Supplementary Material

# Supplementary Tables

**Supplementary Table 1** Main research content of the top 25 references with the strongest citation bursts

| Title | Key points | Strength | Year | Begin | End |
| --- | --- | --- | --- | --- | --- |
| Effects of blood pressure lowering with perindopril and indapamide therapy on dementia and cognitive decline in patients with cerebrovascular disease | To determine whether blood pressure lowering would reduce the risks of dementia and cognitive decline among individuals with cerebrovascular disease | 15.76 | 2003 | 2004 | 2008 |
| The prevention of dementia with antihypertensive treatment: new evidence from the Systolic Hypertension in Europe (Syst-Eur) study | To refine the estimates of the long-term effects of antihypertensive therapy on the incidence of dementia | 15.69 | 2002 | 2004 | 2007 |
| Dyskinesias, tics, and psychosis: Issues for the next Diagnostic and Statistical Manuel of Mental Disorders | To highlight an issue concerning the criteria for tic disorders, and how this might affect classification of dyskinesias in psychotic spectrum disorders | 11.86 | 2011 | 2011 | 2013 |
| Midlife cardiovascular risk factors and risk of dementia in late life | To evaluate if midlife cardiovascular risk factors are associated with risk of late-life dementia in a large, diverse cohort | 15.14 | 2005 | 2006 | 2010 |
| Aggregation of vascular risk factors and risk of incident Alzheimer disease | To explore the association of the aggregation of vascular risk factors with AD | 14.19 | 2005 | 2006 | 2010 |
| The age-dependent relation of blood pressure to cognitive function and dementia | To investigate the age-related relationship between blood pressure, cognitive function, and dementia | 14.19 | 2005 | 2006 | 2010 |
| Antihypertensive medication use and incident Alzheimer disease: the Cache County Study | To examine the relationship of AH medication use with incidence of AD among the elderly population of Cache County, Utah, and to examine whether the relationship varies with different classes of AH medications | 13 | 2006 | 2006 | 2011 |
| Global prevalence of dementia: a Delphi consensus study | To determine dementia prevalence for each world region | 11.68 | 2005 | 2007 | 2010 |
| Incident dementia and blood pressure lowering in the Hypertension in the Very Elderly Trial cognitive function assessment (HYVET-COG): a double-blind, placebo controlled trial | To assess the risks and benefits of treatment of hypertension in elderly patients and included an assessment of cognitive function | 18.09 | 2008 | 2009 | 2013 |
| Less Alzheimer disease neuropathology in medicated hypertensive than nonhypertensive persons | To test the hypothesis that use of antihypertensive medication is associated with lower Alzheimer disease (AD) neuropathology | 11.72 | 2009 | 2010 | 2013 |
| Use of angiotensin receptor blockers and risk of dementia in a predominantly male population: prospective cohort analysis | To investigate whether angiotensin receptor blockers protect against Alzheimer's disease and dementia or reduce the progression of both diseases | 19.56 | 2010 | 2011 | 2015 |
| Vascular contributions to cognitive impairment and dementia: a statement for healthcare professionals from the american heart association/american stroke association | To gain a better understanding of VCI and dementia, prevention, and treatment | 16.67 | 2011 | 2012 | 2016 |
| Associations of anti-hypertensive treatments with Alzheimer's disease, vascular dementia, and other dementias | To investigate whether angiotensin II receptor blockers (ARBs) and angiotensin converting enzyme inhibitors (ACE-Is) are more strongly associated with Alzheimer's disease (AD), vascular dementia (VaD), and other dementias, than other anti-hypertensive drugs | 12.01 | 2011 | 2012 | 2016 |
| Neurovascular pathways to neurodegeneration in Alzheimer's disease and other disorders | To examine mechanisms of BBB dysfunction in neurodegenerative disorders, notably Alzheimer's disease, and highlights therapeutic opportunities relating to these neurovascular deficits | 12.22 | 2011 | 2013 | 2016 |
| Hypertension induces brain β-amyloid accumulation, cognitive impairment, and memory deterioration through activation of receptor for advanced glycation end products in brain vasculature | To investigate the mechanisms involved in Alzheimer disease of hypertensive mice | 11.68 | 2012 | 2013 | 2017 |
| Potential for primary prevention of Alzheimer's disease: an analysis of population-based data | To provide specific estimates of preventive potential by accounting for the association between risk factors | 12.9 | 2014 | 2015 | 2019 |
| Hypertension enhances Aβ-induced neurovascular dysfunction, promotes β-secretase activity, and leads to amyloidogenic processing of APP | To examine the impact of HTN on the vascular dysfunction induced by Aβ to gain insight into whether HTN may interact synergistically with Aβ to worsen the ability of cerebral blood vessels to regulate blood flow | 12.08 | 2016 | 2017 | 2020 |
| Dementia prevention, intervention, and care | To summarize the prevention, intervention, and care of dementia | 16.55 | 2017 | 2018 | 2023 |
| Association Between Midlife Vascular Risk Factors and Estimated Brain Amyloid Deposition | To determine if midlife vascular risk factors are associated with late-life brain amyloid deposition, measured using florbetapir positron emission tomography (PET) | 14.28 | 2017 | 2018 | 2023 |
| Impact of Hypertension on Cognitive Function | To provide an appraisal of the contribution of hypertension to age-related cognitive dysfunction | 13.15 | 2016 | 2018 | 2021 |
| NIA-AA Research Framework: Toward a biological definition of Alzheimer's disease | The research framework focuses on the diagnosis of AD with biomarkers in living persons | 14.69 | 2018 | 2019 | 2023 |
| Effect of Intensive vs Standard Blood Pressure Control on Probable Dementia: A Randomized Clinical Trial | To evaluate the effect of intensive blood pressure control on risk of dementia | 24.09 | 2019 | 2020 | 2023 |
| Association of Midlife to Late-Life Blood Pressure Patterns With Incident Dementia | To examine the association of midlife to late-life BP patterns with subsequent dementia, mild cognitive impairment, and cognitive decline | 12.7 | 2019 | 2020 | 2023 |
| Dementia prevention, intervention, and care: 2020 report of the Lancet Commission | To specify policy and individual changes to delay the onset of cognitive impairment and dementia and better ways to support and treat people with dementia and their families and to improve their quality of life | 28.22 | 2020 | 2021 | 2023 |
| Antihypertensive medications and risk for incident dementia and Alzheimer's disease: a meta-analysis of individual participant data from prospective cohort studies | To explore whether taking specific antihypertensive drugs (AHM) can reduce the risk of dementia | 12.6 | 2020 | 2021 | 2023 |
